# Assessment of ecological fidelity of human microbiome-associated mice in observational studies and an interventional trial

**DOI:** 10.1128/mbio.01904-25

**Published:** 2025-09-25

**Authors:** Matthew K. Wong, Eric Armstrong, Alya A. Heirali, Pierre H. H. Schneeberger, Helen Chen, Kyla Cochrane, Keith Sherriff, Emma Allen-Vercoe, Lillian L. Siu, Anna Spreafico, Bryan Coburn

**Affiliations:** 1 Department of Immunology, University of Toronto 7938, Toronto, Canada; 2 Toronto General Hospital Research Institute, University Health Network 7989, Toronto, Canada; 3 Department of Medical Parasitology and Infection Biology, Swiss Tropical and Public Health Institute 30247, Allschwil, Switzerland; 4 University of Basel 27209, Basel, Switzerland; 5 National Cancer Institute, National Institutes of Health 2511, Bethesda, Maryland, USA; 6 Nubiyota LLP, Guelph, Canada; 7 University of Guelph 3653, Guelph, Canada; 8 Tumor Immunotherapy Program, Princess Margaret Cancer Centre, University Health Network 7989, Toronto, Canada; 9 Division of Medical Oncology and Hematology, Princess Margaret Cancer Centre, University Health Network, University of Toronto 7989, Toronto, Canada; University of Maryland School of Medicine, Baltimore, Maryland, USA

**Keywords:** gut microbiome, microbial consortia, human microbiota-associated mice, fecal microbiota transplant

## Abstract

Composition and function of the gut microbiome are associated with diverse health conditions and treatment responses. Human microbiota-associated (HMA) mouse models are used to establish causal links for these associations but have important limitations. We assessed the fidelity of HMA mouse models in recapitulating ecological responses to a microbial consortium using stools collected from a human clinical trial. HMA mice were generated using different routes of consortium exposure, and their ecological features were compared to human donors by metagenomic sequencing. HMA mice resembled other mice more than their respective human donors in gut microbial composition and function, with taxa including *Akkermansia muciniphila* and *Bacteroides* spp. enriched in mouse recipients. A limited repertoire of microbes was able to engraft into HMA mice regardless of route of consortium exposure. In publicly available HMA mouse data sets from four distinct health conditions, we confirmed our observation that a taxonomically restricted set of microbes reproducibly engrafts in HMA mice and observed that stool microbiome composition of HMA mice was more like other mice than their human donor. Our data suggest that HMA mice are limited models for assessing the ecological impact of microbial consortia, with ecological effects in HMA mice being more strongly associated with host species than donor stool ecology or ecological responses to treatment in humans. Comparisons to published studies suggest this may be due to comparatively large host-species effects that overshadow ecological effects of treatments in humans that HMA models aim to recapitulate.

**IMPORTANCE:**

HMA mice are models that better represent human gut ecology compared to conventional laboratory mice and are commonly used to test the effects of the gut microbiome on disease or treatment response. We evaluated the fidelity of using HMA mice as avatars of ecological response to a human microbial consortium, Microbial Ecosystem Therapeutic 4. Our results show that HMA mice in our cohort and across other published studies are more similar to each other than the human donors or inoculum they are derived from and harbor a taxonomically restricted gut microbiome. These findings highlight the limitations of HMA mice in evaluating the ecological effects of complex human microbiome-targeting interventions, such as microbial consortia.

## INTRODUCTION

Many associations have been identified between gut microbial composition and pathological or physiological phenotypes, including health conditions such as obesity (1, 2), diabetes (3), inflammatory bowel disease (4), neurological disorders (5–7), and allergy (8), or therapeutic responses, such as cancer immunotherapy (9–11). Human microbiota-associated (HMA) mouse models have been instrumental in exploring these interactions. HMA mice are generated by fecal microbiota transplant (FMT) of human stool into germ-free (GF) or antibiotic-treated mice to recreate human donor ecology and assess associated pathology and other phenotypes (12). This approach provides a model by which phenotypic differences can be causally attributed to compositional differences in or between the human donors’ gut microbiomes and provides a platform for interrogating mechanism with experimental strategies that would be impractical or impossible in humans.

Although HMA mouse models have become a common tool for inferring causal effects of the gut microbiome in clinical studies, they have been subject to criticism and scrutiny (12–15). Dietary and environmental differences between host species contribute to intrinsic ecological differences in the gut microbiome of humans and mice (16, 17). Host physiology also differs between species, including differences in gastrointestinal tract anatomy (18, 19) and broader host systems like immunity (20, 21) and metabolism (22, 23), which can affect the ability for human-adapted microbes to colonize the murine gut. Complex human microbial communities may therefore only partially engraft in mice, which may affect the validity of phenotype transfer into HMA mice if microbes associated with human phenotypes are excluded.

Microbial consortia are curated communities of microbes that are designed to be a safer and more reproducible alternative to fecal microbiota transplant (24). Therapeutic mechanisms of disease modification by consortia have been assessed in mouse models of a diversity of conditions in which the microbiome has been causally implicated, such as *Clostridioides difficile* infection (25) and non-infectious conditions such as cancer (26, 27). A recent clinical trial, MET4-IO (28), tested for the safety and engraftment of Microbial Ecosystem Therapeutic 4 (MET4), a microbial consortium designed for combination with cancer immunotherapy. This presented an opportunity to study the ecological fidelity of HMA mouse models as “avatars” of human ecological responses to a microbiome-targeting intervention.

Here, we generated HMA mice using stool from donors that participated in MET4-IO with the purpose of assessing the concordance of ecological responses to a microbial consortium between HMA mice and their corresponding donors. Ecological recapitulation of human microbiomes in HMA mice from published data sets was then performed to determine how generalizable our results were. The goals of this study were to assess how well HMA mice represented human ecological changes that occur during therapeutic administration of microbial consortia and ascertain their suitability as experimental avatars in this context.

## MATERIALS AND METHODS

### Study cohort and sample collection

Individuals with solid tumors were enrolled in an early-phase clinical study that assessed the safety, tolerability, and engraftment of MET4, a novel microbial consortium (28). Participants from “cohort B” were the primary focus of this study, consisting of immune checkpoint inhibitor (ICI)-naïve participants who were stratified into MET4 + standard-of-care ICI (anti-PD-1 alone or in combination with anti-CTLA-4) or ICI alone (no MET4) treatment groups. Further details of the trial are detailed in the MET4-IO trial publication (28).

Paired participant stool samples were collected from MET4-IO cohort B participants from both MET4-treated and control groups. Samples were collected 3–4 weeks after initiation of ICI but prior to MET4 (T0) and 3–4 weeks after initiation of MET4, while on ICI (window +2 weeks [T2]). If a T2 sample was not available, an end-of-therapy sample was used if it was collected no more than 2 weeks after the scheduled timing for T2. Stools were frozen at −80°C.

### MET4 consortium production and composition

MET4 consists of 30 individually cultured bacterial isolates chosen based on association with ICI response (9–11). Bacterial strains were encapsulated together and assessed for consistency between drug batches. The taxonomic composition of MET4 has previously been reported (28) and is included in Table S1, along with taxonomic annotations used in our analyses.

### Stool processing

Phosphate-buffered saline (PBS) + 20% glycerol was degassed in an anaerobic chamber overnight prior to stool processing. Stools were thawed and resuspended at 10 mL/g in degassed PBS + 20% glycerol to make a fecal slurry. Slurries were passed through a 300 µm Whirl-Pak filter bag (Nasco) under anaerobic conditions to remove stool particulates. Filtered fecal slurries were frozen at −80°C until use.

### Human microbiota-associated mice

To generate the HMA mice, germ-free mice were gavaged with 250 µL of thawed human donor fecal slurries and caged separately based on donor stool received. No mice generated with different donor stools were co-housed. Extra slurry was lathered onto animal fur and cage bedding. Cages were kept in germ-free conditions for 2 weeks before being moved to specific pathogen-free (SPF) housing conditions, where mice were not handled for a week to allow the gut microbiome to stabilize. Stools were collected at baseline, 1 week after SPF entry, and 3–4 weeks after SPF entry.

Using this method, paired pre-MET4-/post-MET4-treated HMA mice were generated with two “routes” of MET4 exposure. For the first, which we termed the “human-treated route,” mice were generated using paired stool samples obtained at T0 and T2 from six study participants (participants B001, B002, B004, B005, B012, and B018), i.e., before and after MET4 administration to those participants. For the alternative method, which we termed the “mouse-treated route” (MTR), HMA mice were generated using T0 (pre-MET4 treatment) stools from participants B004 and B005 (both with increases of >5 MET4 taxa by >10 fold after treatment). Once the gut microbiome was stabilized, mice were treated directly with MET4. One week after SPF entry, MET4 was processed and diluted in 5 mL of degassed PBS anaerobically. Two hundred fifty microliters of MET4 was gavaged into mice for three consecutive days and every 3 days for 2 weeks thereafter. Stools were collected at baseline, SPF entry, and 2 weeks post-MET4 initiation.

### Metagenomic sequencing

DNA was extracted from donor fecal slurries and mouse stools using Qiagen’s DNeasy Powersoil Pro kit. Illumina’s DNA Prep kit was used to prepare libraries from these samples. Nextera 96-well CD Indexes (Illumina) were used to index each sample before sequencing on an Illumina MiniSeq with a MiniSeq High Output Reagent Kit (Illumina) to obtain approximately 500,000 reads/sample (25 million total reads).

### Sequence data processing

Sequence quality was assessed with FastQC v.0.11.9 (29). As the quality was high, no sequence trimming was performed. Nextera adapters were trimmed with Trimmomatic v.0.39 (30). Human, phiX, and mouse reads were removed with KneadData v.0.7.2(31). Taxa were identified from cleaned reads using Metaphlan v.4.0.6 using default settings (32, 33). Functional metagenomic profiles were generated with HUMAnN3 v3.9(34) with default parameters. Gene pathway abundances were normalized to copies per million (CPM) for downstream analyses.

### Microbiome statistical analysis

For all analyses, mice generated from the same donor stool were averaged together to avoid pseudo-replication (13). Mice were therefore treated as technical replicates. MET4 taxonomic annotations used for analyses are listed in Table S1. Percent relative abundance (RA) was compared between stools collected from mice generated from each donor-mouse pair at T0 and T2 timepoints. Percent RAs were averaged for mice generated with a single donor stool to avoid pseudo-replication (12, 13). Bray-Curtis dissimilarity matrices were generated with the “vegdist” function in the vegan package in R (35), using species-level taxonomy tables as input. Pairwise dissimilarities were manually extracted from the resulting dissimilarity matrix for group-wise comparisons. For MaAsLin2 analysis, taxonomic tables were filtered by removing species that contributed less than 0.1% relative abundance in one sample. The MaAsLin2 package v.1.7.3 (36) was used to identify taxa that were enriched in humans vs mice. The fixed effect was host species (i.e., human vs mouse). For models that included repeated measurements from individual participants, participant ID and timepoint were included as random effects. Default MaAsLin2 analysis parameters were used, and false discovery rate was used to control for multiple comparisons. A *q* value of 0.05 was considered significant.

### Mega-analysis on human microbiota-associated mouse studies

Four HMA mouse studies across distinct health states (inflammatory bowel disease, Parkinson’s disease, colorectal cancer, and autism spectrum disorder) were selected for a mega-analysis (4, 6, 7, 37). Studies were selected based on public availability of raw 16S rRNA sequencing data for human donors and mice and appropriate study design (i.e., FMT of human donor stool to recipient mice). Raw sequencing data were analyzed with QIIME2 v.2024.2 (38). Quality filtering and denoising were performed with DADA2 (39). Taxonomic assignment of sequences was performed using a naïve Bayes classifier trained on the SILVA 132 99% operational taxonomic unit database (40, 41). Amplicon sequence variant tables were defined at the genus level. For Pearson correlation coefficient comparisons, a Fisher *z*-transformation was performed on coefficients prior to analysis. Percent engraftment was calculated for each taxon by dividing total human-mouse pairs with the taxon present in both by total human donors with the taxon present overall. For engraftment plots, only taxa engrafting in >4 mice were included. Bray-Curtis dissimilarity was generated and analyzed as above.

## RESULTS

### Post-treatment stool composition differs in human donors and mouse recipients

**Fig 1:**
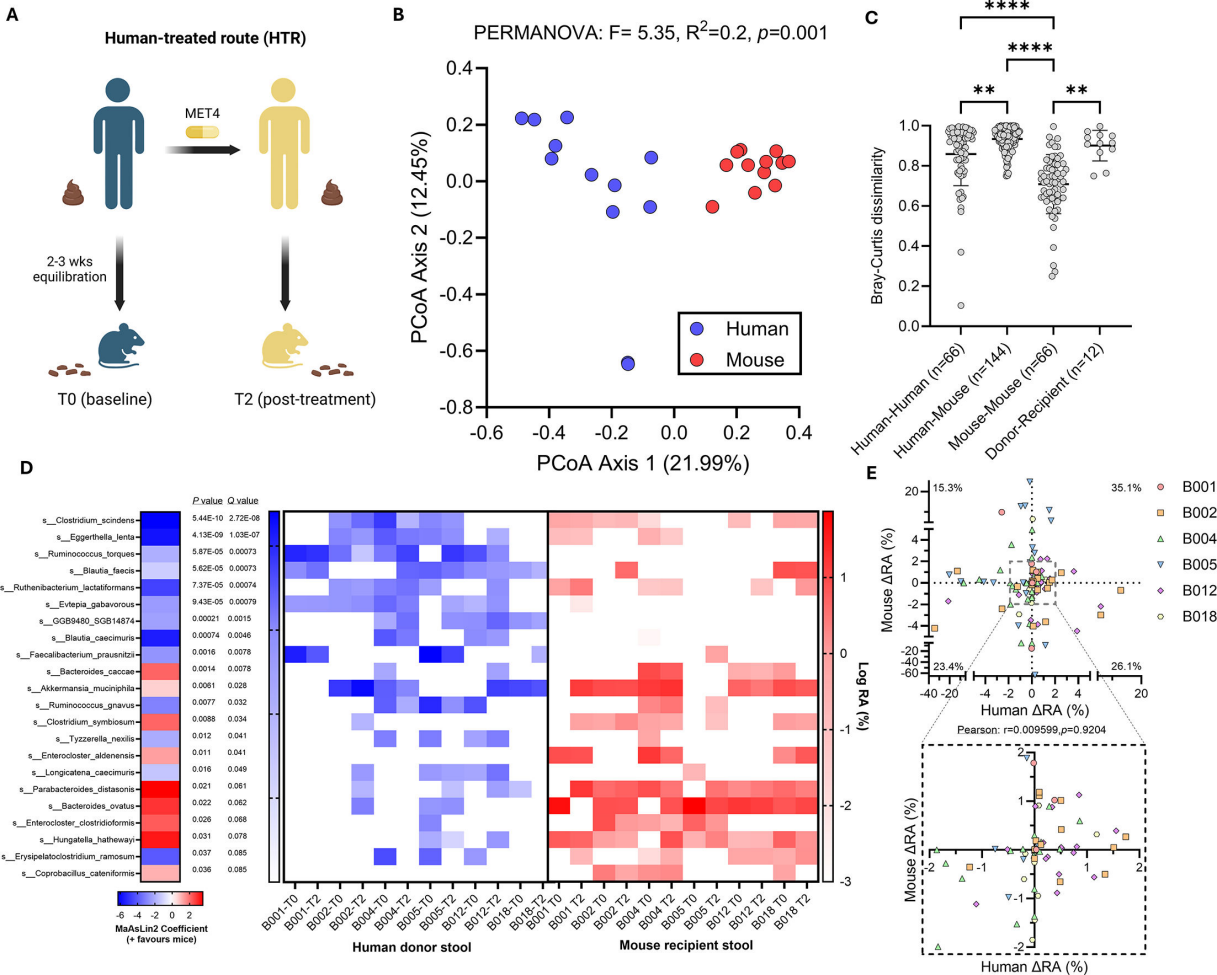
Different gut microbiota engraft in mouse recipients compared to human donors. (**A**) Flowchart depicting generation of HMA mice. (**B**) Principal coordinate analysis (PCoA) plot for Bray-Curtis dissimilarity between human donor and mouse stool composition. Each dot is an individual human donor sample or an average of mouse recipient samples from the same donor. PERMANOVA compares species composition. (**C**) Bray-Curtis dissimilarity for all comparisons between donors, mice, and between donor and respective recipients. Lines indicate the mean, and whiskers are the standard deviation. Kruskal-Wallis test followed by Dunn’s multiple comparison test was performed. (**D**) Association between species and relative abundance (RA) of stool taxa in human donors and recipient mice, including donor ID and treatment timepoint as random effects. Blue shading indicates taxa enriched in mice, while red indicates taxa enriched in humans. Statistics and coefficients determined with MaAsLin2. Heat map displays the log % relative abundance of corresponding taxa in individual human and mouse samples. Zero values are arbitrarily set to −3. (**E**) Scatter plot of delta % RA for stool taxa from baseline to post-MET4 timepoints in human donors and mouse recipients. Each dot represents an individual taxon, and only taxa overlapping between humans and mice were included. Percentage of overlapping taxa is shown for each quadrant. Pearson’s correlation test was performed. **P < 0.01; ****P < 0.0001.

We first sought to assess the reproducibility of human ecology in HMA mice generated using six sets of paired stools collected pre-MET4 (on ICI, before MET4) and post-MET4 (on ICI and on MET4) treatment from MET4-IO participants (Fig. 1A). Stools from mouse recipients were sequenced alongside human donor stools to identify differences in stool composition between donor-recipient pairs. Human donor stools were represented by a large proportion of Bacillota with some Actinomycetota and Bacteroidota, while mouse recipient stools were dominated by Bacteroidota, Bacillota, and Verrucomicrobiota (Fig. S1). Stool microbiomes of HMA mice were more compositionally similar to mice from other donors than the stool microbiome of their human donor (permutational multivariate analysis of variance [PERMANOVA], *F* = 5.35, *R*^2^ = 0.2; Fig. 1B and C; Fig. S2). Dispersion differed between human and mouse groups, indicating lower variance in mouse recipient microbiomes compared to human donors (permutational multivariate analysis of dispersion [PERMDISP], *F* = 9.31, *P* = 0.0058).

When controlling for donor and timepoint, specific taxa were found to be enriched in either mouse or human stools, suggesting engraftment bias of distinct taxa, depending on host species (Fig. 1D). *Clostridium scindens*, *Eggerthella lenta*, and *Ruminococcus torques* were enriched in human donors, while *Bacteroides caccae*, *Akkermansia muciniphila*, and *Clostridium symbiosum* increased in mouse recipients (Fig. 1D). T0/T2 differences in percent relative abundance of overlapping taxa between donor-recipient pairs revealed poor correlativity, indicating that taxonomic changes after treatment in humans were not recapitulated in corresponding HMA mice (Fig. 1E). Only a subset of taxa in donor stools appeared in the stool of recipient mice, with variable engraftment (5.13%–34.69%) across donors (Fig. S3).

### Metabolic pathways in the gut microbiome differ between humans and HMA mice

We next investigated metagenomic composition of the gut microbiome to determine whether functional gene profiles also differed between host species. Like taxonomic composition, stool metagenomic composition in mice differed from their human donors (PERMANOVA, *F* = 8.17, *R*^2^ = 0.27; Fig. S4). In human stools, carbohydrate metabolism (lactose and galactose degradation) and a variety of anabolic processes, including bacterial component (cell wall precursor: UDP-N-acetyl-D-glucosamine biosynthesis 1, LPS: O-antigen building block biosynthesis, *Escherichia coli*), nucleotide (inosine-5′phosphate biosynthesis III), and amino acid biosynthesis (L-lysine biosynthesis I, superpathway of L-alanine biosynthesis), were elevated compared to mouse recipients (Fig. S5).

In contrast, HMA mice displayed increased amino acid degradation (L-histidine degradation I/III), cofactor biosynthesis (vitamin B1: thiamine diphosphate biosynthesis I, vitamin B6: pyridoxal 5′-phosphate biosynthesis and salvage, and vitamin B9: 6-hydroxymethyl-dihydropterin diphosphate biosynthesis I), and glycoside/carbohydrate degradation (beta-D-glucuronosides degradation and L-rhamnose degradation I) compared to their human donors (Fig. S5). Taxa elevated in mice (Fig. 1D), especially *Bacteroides* and *Parabacteroides* spp., are associated with many of these pathways, including histidine degradation (42), vitamin biosynthesis (43), host-derived glucuronide degradation (44), and rhamnose metabolism (45), which match the functional profile observed in these mice.

These differences in gene pathway abundance between host species suggest that the HMA mouse gut microbiome in our cohort is not only compositionally dissimilar but also possesses different functional capabilities (metagenomes) compared to their human donor microbiomes.

### Donor-dependent differences in HMA mouse gut ecology exist after treatment exposure despite similarities between HMA mice

**Fig 2:**
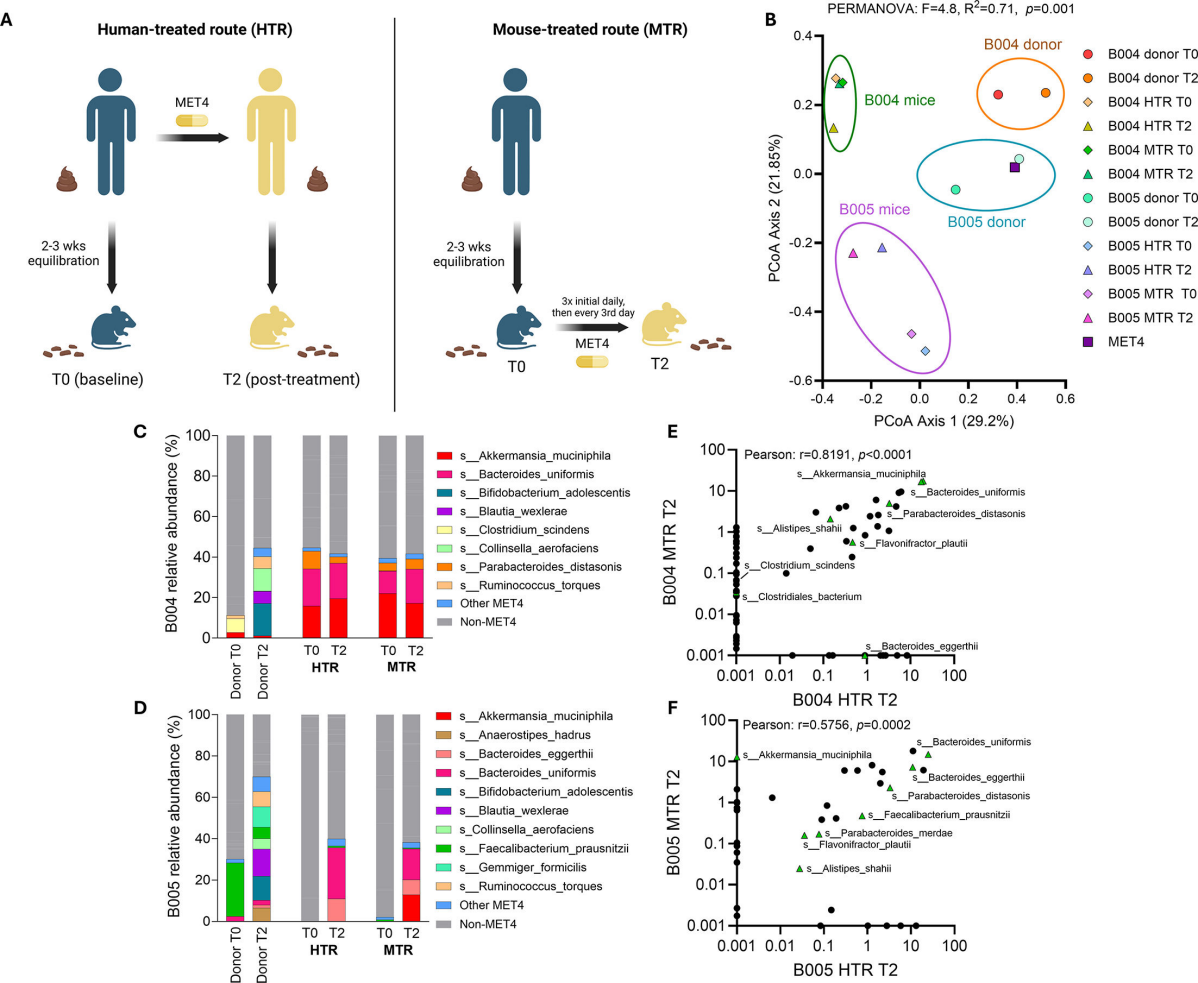
Analysis of the gut microbiome following different treatment exposure routes in germ-free mice. (**A**) Diagram describing the two routes of treatment exposure in this study. Stools were collected at the indicated timepoints. (**B**) Principal coordinate analysis (PCoA) plot of Bray-Curtis dissimilarity for MET4, donor stools, and stool from mouse recipients receiving donor FMT and/or direct MET4 administration. Each dot represents an individual human donor sample, an average of MET4 samples sequenced, or an average of mouse recipient stools from the same donor and treatment route. Diamonds represent T0 timepoints; triangles represent T2; and squares represent MET4. PERMANOVA compares MET4, donor stool, and mouse recipient stool composition. (**C and D**) Average relative abundance (%) histograms for stools from mice receiving post-MET4 (T2) donor stool from B004 (**C**) and B005 (**D**) or MET4 directly. Donor stool composition is included as a comparator. Non-MET4 taxa are in grayscale and unlabeled. “Other” consists of taxa with <5% relative abundance in every sample. (**E and F**) Scatter plots showing log scale % relative abundance of gut taxa in B004 (**E**) and B005 (**F**) mice compared between routes of exposure at T2. Each point represents a different taxon. MET4 taxa are represented by green triangles.

Changes in the gut microbiome of HMA mice receiving MET4-treated human (T2) stool include all aggregate exposures experienced by the human donor (including diet, medical interventions, and other factors) and may not be solely attributable to consortium exposure. We therefore evaluated ecological changes after direct inoculation of MET4 to assess ecological effects in the absence of these potential confounders. We administered MET4 directly to HMA mice (MTR) after prior gavage with T0 donor stool to compare taxonomic stool composition to mice receiving T2 donor stool (human-treated route [HTR], Fig. 2). We generated these mice with stool from two donors (B004 and B005) in which a significant proportion of MET4 engrafted (Fig. S6A).

To assess differences in stool composition between MTR and HTR routes, Bray-Curtis dissimilarity was calculated between mouse samples, donor stools, and the MET4 consortium itself. Distinct compositional clusters were observed for human donors and mouse recipients that were donor specific (PERMANOVA, *F* = 4.8, *R*^2^ = 0.71; Fig. 2B). For each set of mice generated from the same donor, mouse microbial communities were compositionally similar regardless of the route of MET4 exposure. When comparing Bray-Curtis dissimilarity, MET4 and donor stools were highly dissimilar to MTR stools, while MET4-exposed mouse stools were more similar regardless of exposure route (Fig. S6B). Mice receiving B004 donor stool were more compositionally similar to one another than those receiving B005 donor stool (Fig. S6C and D) regardless of the method by which they were generated (Fig. S6C and E), indicating changes in HMA mouse microbial ecology after treatment depended on the donor.

### Compositional similarities in HMA mice generated after therapeutic microbial consortium exposure are driven by engraftment of a limited set of taxa

To identify donor-dependent differences in ecology, we compared the percent relative abundance of MET4 taxa between donor stools and routes of treatment exposure for each group of recipient mice. Mice receiving stool from B005, but not B004, displayed increases in MET4 relative abundance by both exposure routes (Fig. 2C and D; Fig. S7A and B). The dominant engrafting consortium microbes in B005 recipient mice resembled those in B004 mice, which displayed engraftment of MET4 taxa at T0, indicating the presence of these taxa even without MET4 exposure in B004 mice. There was a positive correlation in the relative abundance of taxa present in HMA mice regardless of the MET4 exposure route (Fig. 2E and F; Fig. S7C and D). *Alistipes shahii*, *Parabacteroides distasonis*, *Bacteroides uniformis*, *Bacteroides eggerthii*, and *Flavonifractor plautii* were present in post-treatment mice regardless of donor stool or treatment exposure route, suggesting that engraftment of MET4 microbes may be taxonomically restricted in HMA mice.

### Engraftment of a restricted set of taxa is reproducible in HMA mice generated from donors with a variety of health states

To assess whether taxonomically restricted engraftment of donor microbes in HMA mice was unique to our experiments, we analyzed data sets from four studies (Table 1) in which HMA mice were generated from phenotypically diverse human health states, including colon cancer, autism spectrum disorder, inflammatory bowel disease, and Parkinson’s disease (4, 6, 7, 37). Stool microbiome composition from human donors and mouse recipients was compared after processing and analyzing publicly available sequencing data from these studies through a single analytical pipeline.

**TABLE 1:** Studies included in HMA mouse mega-analysis

Study	Condition	Sequencing type	# of donor-mouse groups used
Britton et al. (4)	Intestinal bowel disease	16S	15
Sampson et al. (6)	Parkinson’s disease	16S	12
Baxter et al. (37)	Colorectal cancer	16S	6
Sharon et al. (7)	Autism spectrum disorder	16S	8

**Fig 3:**
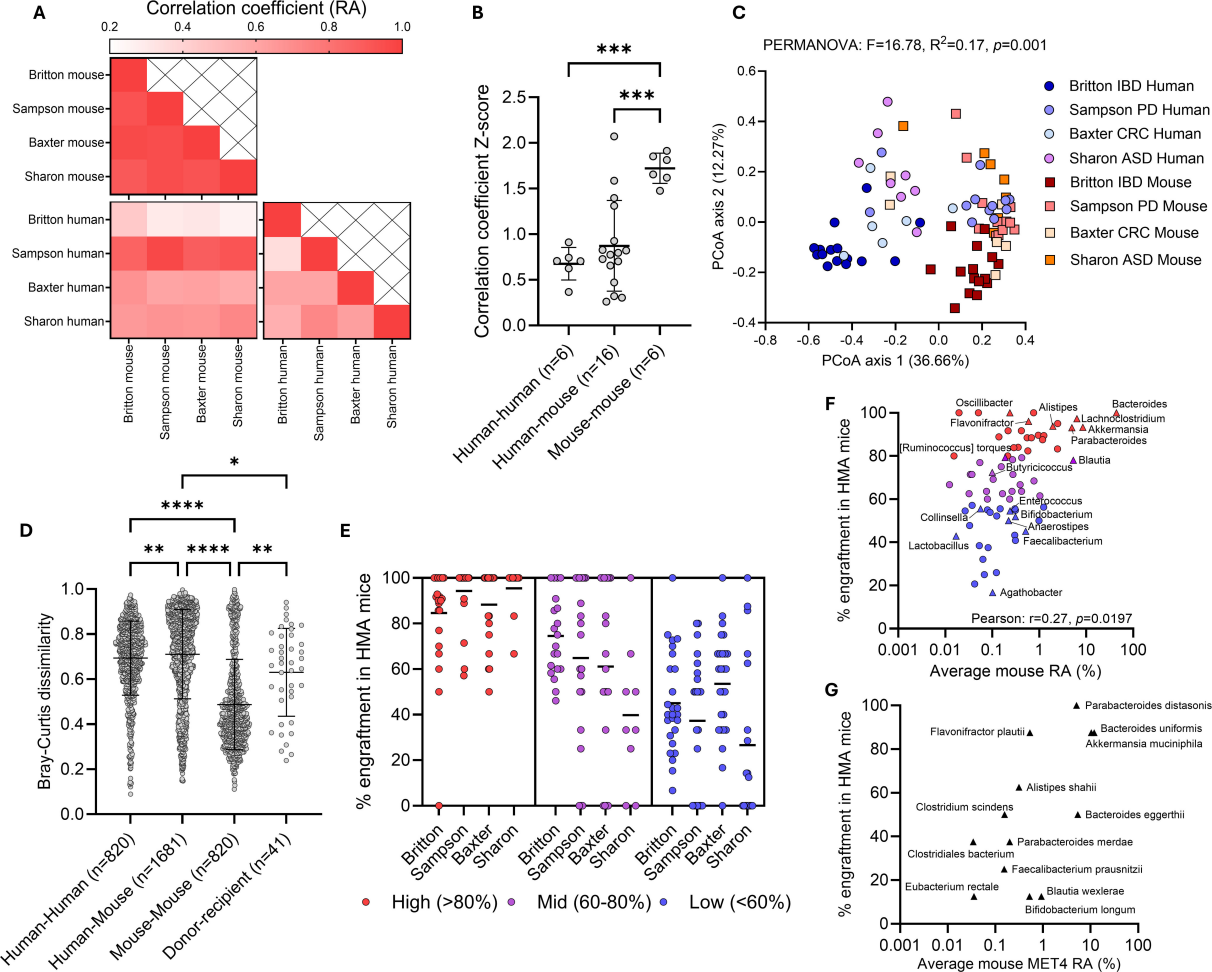
HMA mice engraft specific taxa more consistently. (**A**) Heat map displaying correlation coefficient for relative abundance (RA) of all taxa between studies and species. (**B**) Comparison of *z*-transformed correlation coefficients by analysis of variance followed by Tukey’s multiple comparison test. (**C**) Principal coordinate analysis plot (PCoA) of Bray-Curtis dissimilarity comparing HMA mice to human donors from different studies. Each dot represents a human donor or an average of mouse recipient stools. Circles represent humans; squares represent mice. PERMANOVA compares species composition. (**D**) Bray-Curtis dissimilarity for all comparisons between donors, mice, and between donor and respective recipients. Kruskal-Wallis test followed by Dunn’s multiple comparison test was performed. (**E**) Percent (%) engraftment of taxa from human donors into recipient mice in published data sets. Only taxa engrafting in >4 mice are included. Each dot represents % engraftment of one taxon in individual studies, and lines represent the mean across taxa within the study. Taxa were stratified into high, medium, or low engrafters based on average percent engraftment across studies. (**F and G**) Scatter plots of percent engraftment against average mouse RA across HMA mouse studies (**F**) or average mouse RA of MET4 taxa in mice exposed to MET4 (**G**). Each dot represents an individual taxon. MET4 taxa are labeled and are represented by triangles. Colored taxa in panel **F** correspond to high, medium, or low engraftment from panel **E**. Lines represent the mean and whiskers represent the standard deviation in all figures. ASD, autism spectrum disorder; CRC, colorectal cancer; IBD, intestinal bowel disease; PD, Parkinson’s disease. *P < 0.05; **P < 0.01; ***P < 0.001; ****P < 0.0001.

As we observed in our experiments, microbiota in HMA mice generated from diverse populations in unrelated studies were more alike to one another than they were to the human donors from which they were derived (Fig. 3A through D). Correlation of taxonomic relative abundances between human donors and mouse recipients varied by study (range: 0.439–0.969, Fig. 3A). However, the degree of agreement between mouse-mouse pairs (mean: 0.935) was higher than for human-mouse pairs (mean: 0.634) and human-human pairs (mean: 0.580), even when mice were derived from donors from different studies (range: 0.901–0.957, untransformed coefficients shown here; Fig. 3B; Fig. S8).

Bray-Curtis dissimilarity analysis between human donors and mouse recipients from each study revealed distinct clustering of human stools from mouse stools across cohorts (PERMANOVA, *F* = 16.78, *R*^2^ = 0.17; Fig. 3C) and differences in dispersion between species (PERMDISP, *F* = 31.63, *P* < 0.0001), indicating lower variance in HMA mouse gut microbiomes as we observed in our cohort. Comparison of Bray-Curtis dissimilarities between species presented greater similarity in HMA mice across studies compared to the human donors they were generated from (Fig. 3D). Notably, one human cohort (Parkinson’s disease [6) displayed strong correlations with cross-study mouse relative abundances (Fig. 3A) and clustered closely with HMA mouse microbiomes (Fig. 3C), suggesting this high degree of similarity was from a human cohort with stool samples that already resembled HMA mice ecology.

To assess whether engraftment was a feature of the taxon rather than the experiment, taxa in each study were stratified into high (>80%), medium (60–80%), or low (<60%) engrafters based on their average percent engraftment across HMA mouse studies. “High” engrafters demonstrated concordance across studies (i.e., were high engrafters in all studies), with moderate and low engrafters demonstrating greater heterogeneity within and between studies (Fig. 3E). Within the high engraftment taxa, *Bacteroides* was the most abundant and consistent engrafter amongst all microbes, with all donors transferring this taxon into their corresponding recipient mice when it was present in the donor (Fig. 3F; Table S2). Among MET4 taxa, *Lachnoclostridium*, *Flavonifractor*, *Alistipes*, *Akkermansia*, and *Parabacteroides* engrafted into mice in over 85% of donor-recipient pairs, while *Collinsella*, *Enterococcus*, *Bifidobacterium*, *Anaerostipes*, *Faecalibacterium*, and *Lactobacillus* engrafted in less than 60% of donor-recipient pairs (Fig. 3F; Table S2). Percent engraftment into HMA mice was positively correlated with average relative abundance in these mice, suggesting taxa that frequently engraft may also dominate the gut microbiome of mouse recipients (Fig. 3F).

Notably, *Parabacteroides distasonis*, *Bacteroides uniformis*, *Flavonifractor plautii*, and *Akkermansia muciniphila* were the most consistent MET4 engrafters in our cohort as well, being present in over 80% of mice exposed to MET4 via donor stool or MET4 directly (Fig. 3G; Table S3). Furthermore, *Alistipes shahii*, *Bacteroides eggerthii*, and *Clostridium scindens* were present in over 50% of mice post-MET4 exposure. Of the 13 MET4 taxa with engraftment in our HMA mice, 8/1/3 were high/mid/low engrafters in published studies (Table S3), while 1 taxon was not clearly identifiable based on annotation.

## DISCUSSION

Differences in human and mouse gut ecology exist due to the physiological and environmental differences between species (12, 14, 16–19). HMA mice are thought to circumvent these differences by possessing a “humanized” gut microbiome. In this study, we sought to assess the ecological fidelity of HMA mice as avatars of humans receiving a microbiome-targeting therapy. Stool microbiome composition and functional gene abundance differed between human donors and mouse recipients but were similar across mice irrespective of human donor. A limited repertoire of microbes engrafted into HMA mice, consisting primarily of *Bacteroides*, *Akkermansia*, and *Parabacteroides* spp. This was corroborated in a mega-analysis of four published HMA mouse studies of diverse human health states.

Compositional differences between human donors and recipient mice are generally recognized as limitations of HMA mouse models (12, 14, 46). However, the experimental significance of inter-species engraftment consistency in HMA mouse models is not commonly addressed in individual studies. In our analysis, we observed similarities in engrafting taxa (*Akkermansia muciniphila*, *Bacteroides*, and *Parabacteroides* spp.) across cohorts and health conditions. Interestingly, many of these consistent engrafting taxa are common colonizers of the mouse colon and associated mucosa (47), suggesting that typical mouse gut ecology may be an important experimental constraint in these models. The compositional similarity between HMA mice across studies and the propensity for specific microbes to engraft suggests that intrinsic host species differences may overwhelm or obscure the ecological effects of human donor phenotypes or treatments that studies attempt to causally assess.

Importantly, this does not invalidate phenotypic differences observed in HMA mice. Inoculation of donor stools from different donor phenotypes/treatment groups has been shown to produce differences in HMA mouse phenotypes for a wide variety of conditions (48), including cancer (49), neurological disorders (5, 50, 51), and metabolic conditions (1, 52, 53). However, our observations raise questions about whether these phenotypes are solely attributable to ecological (or microbial functional) differences between host treatments or phenotypes. Our evaluation of the transferability of ecological effect from humans to HMA mice for a microbiome-targeting therapy suggests that causal inferences should be limited in this context.

Our study has important technical limitations. Although we used relatively conventional strategies for generating HMA mice, we used frozen instead of fresh stool samples, which are more likely to selectively affect taxonomic viability (54). The transition from a GF to an SPF environment, although mitigated by a 3 week stabilization period where mice were not handled, could also have led to environmental effects on composition. Factors including differences in human and mouse diet, dissimilarity in host species physiology or immunity, dependencies on other human-adapted taxa not present in the mouse gut, or variability in inoculum viability through processing and in the mouse gastrointestinal tract are also potential modifiers of engraftment (12). Strategies to augment the engraftment of human gut taxa include co-administration of modified humanized diets (55), humanization of mouse immunity (56), or maintaining mice solely in GF conditions. We did not aim to exhaust the possible explanations for the observed taxonomic restriction but merely to characterize it in commonly used experimental conditions. All these factors may have contributed to a gut community that may have been biased toward the taxa we observed. Notably, however, the high degree of concordance in taxonomic composition we observed in HMA mice both within and between studies indicates that if engraftment is influenced by technical factors, these factors are very likely generalizable across diverse study populations, methods, and settings.

Our results highlight the poor ecological fidelity of HMA mice as models of microbial consortium treatment and suggest that host restriction of taxonomic engraftment is a deterministic process. This may limit the utility of these mouse models for attributing causality to the changes induced by microbial consortia when administered to humans and their appropriateness as avatars of human interventional trials targeting the microbiome. While HMA mice remain essential models for mechanistic and reductionist experiments, the reproducibility of taxonomic restriction we observed is an important consideration when designing experiments to assess broad ecological effects, for which HMA mice as they are commonly generated may be inappropriate models.

## Data Availability

Data are vailable upon reasonable request. Metagenomic sequencing data are available in SRA/NCBI under accession number PRJNA1219628. Analysis code is available at https://doi.org/10.5281/zenodo.16640152. Mega-analysis sequencing data are available from their respective sources.

## SUPPLEMENTAL MATERIAL

Supplemental figures mbio.01904-25-s0001.pdf Fig. S1 to S8.

Supplemental legends mbio.01904-25-s0002.docx Supplemental figure and table legends.

Supplemental tables mbio.01904-25-s0003.xlsx Tables S1 to S3.

ASM does not own the copyrights to Supplemental Material that may be linked to, or accessed through, an article. The authors have granted ASM a non-exclusive, world-wide license to publish the Supplemental Material files. Please contact the corresponding author directly for reuse.
