# Supplementary figures and images for: Assessment of ecological fidelity of human microbiome-associated mice in observational studies and an interventional trial
Source: mBio. 2025 Sep 25;16(11):e01904-25. doi: 10.1128/mbio.01904-25 (PMC12607873; doi:10.1128/mbio.01904-25)

**Human donors**

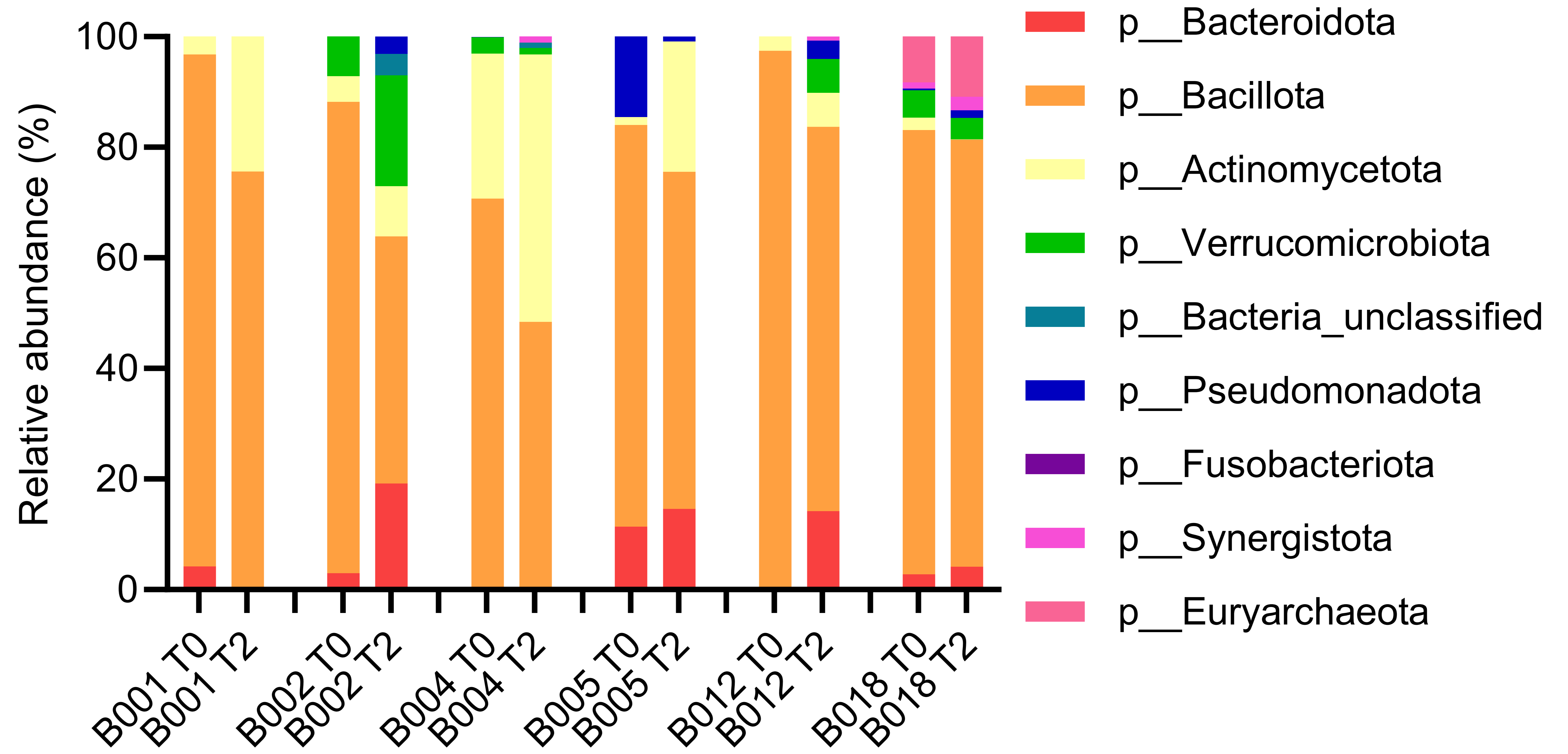

**Mouse recipients**

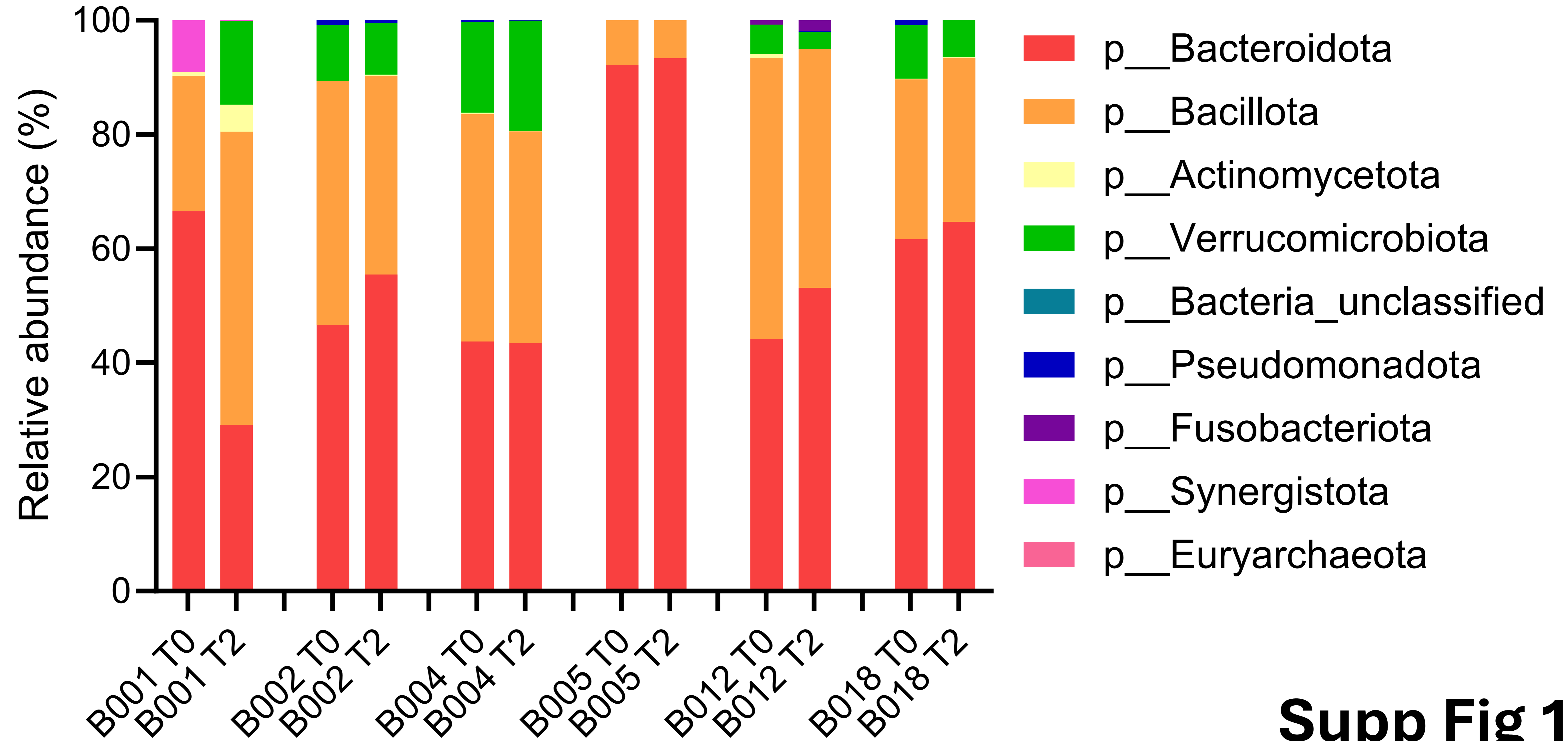

**Supp Fig 1**

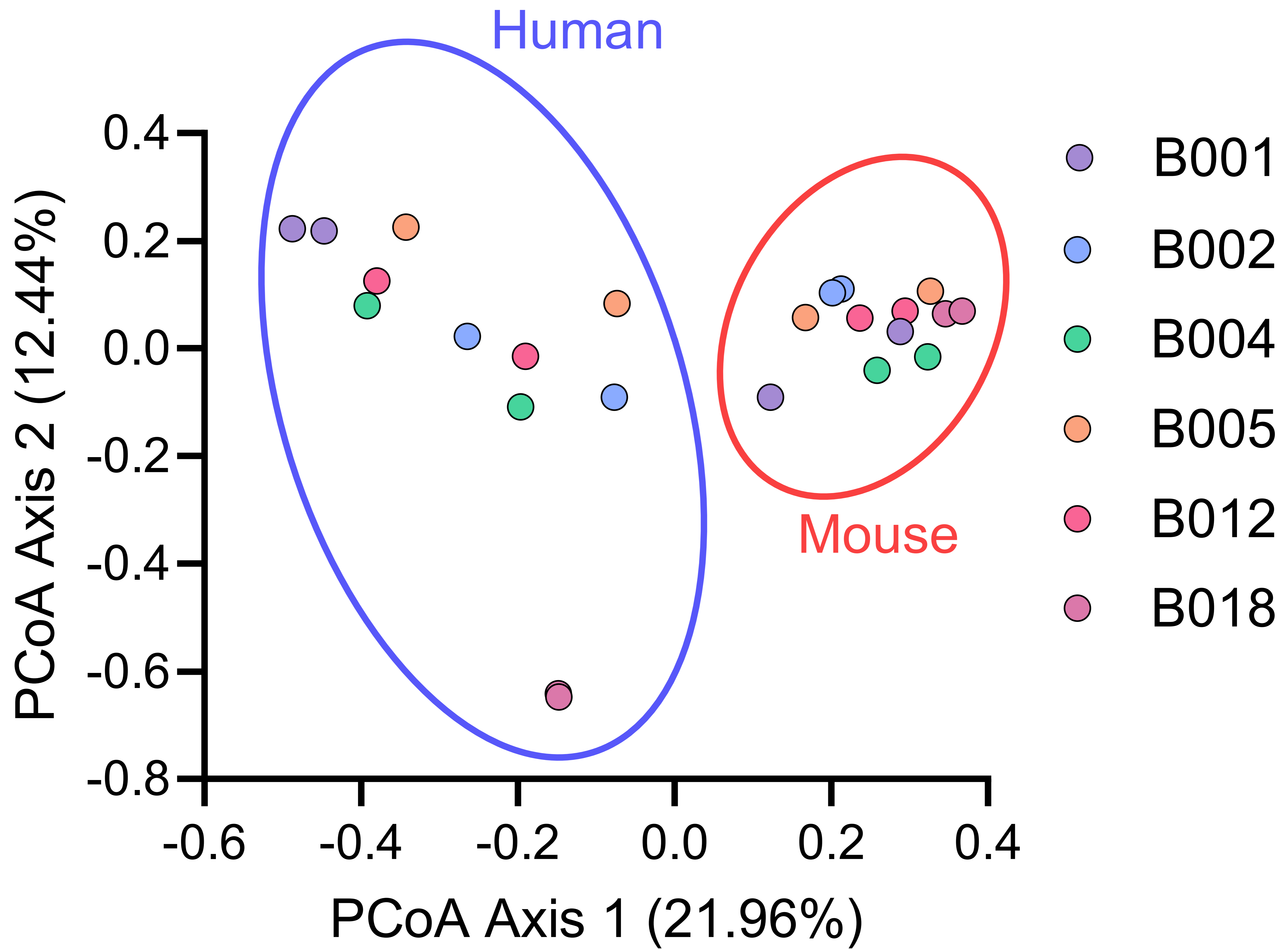

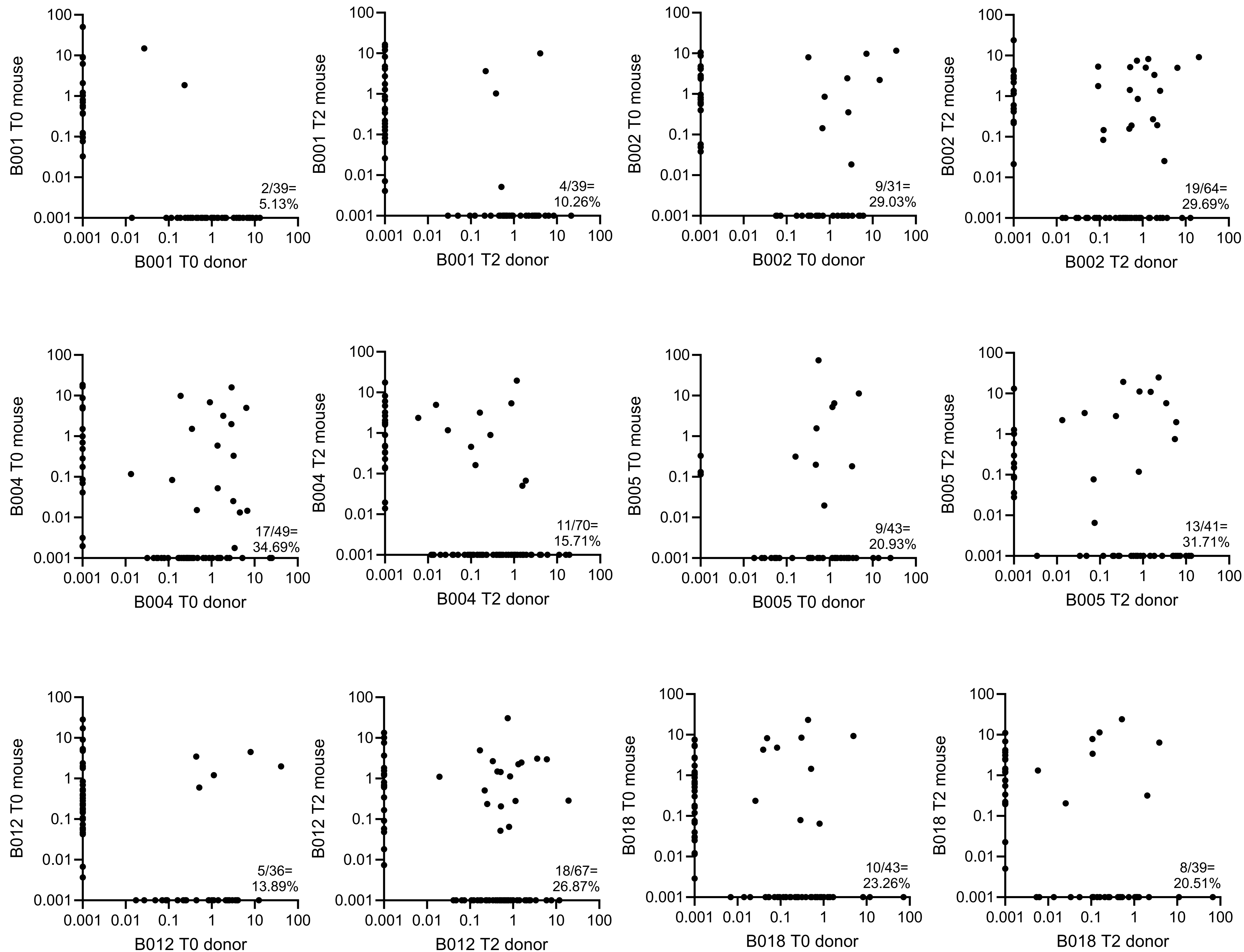

**Supp Fig 3**



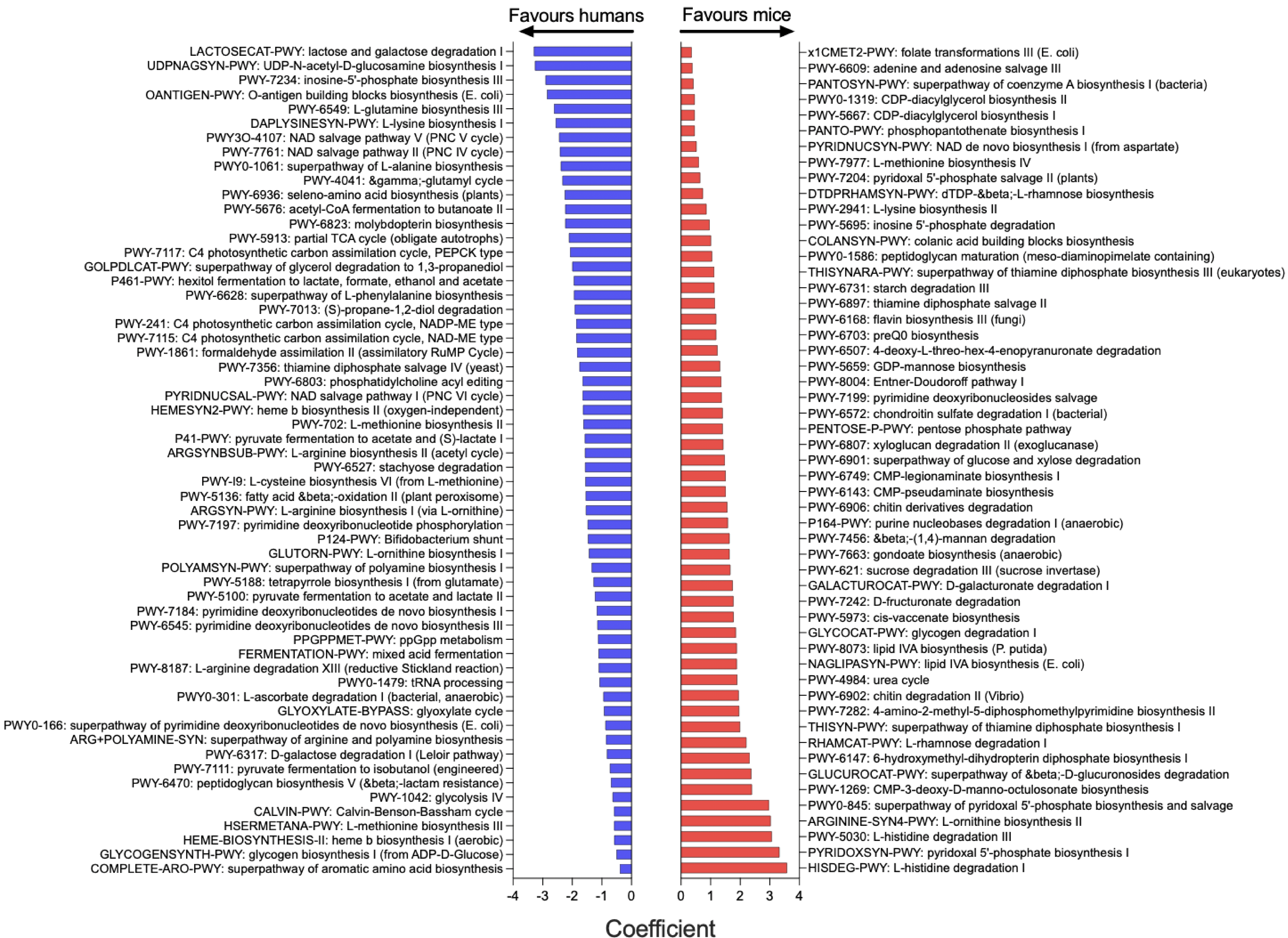

**Supp Fig 5**

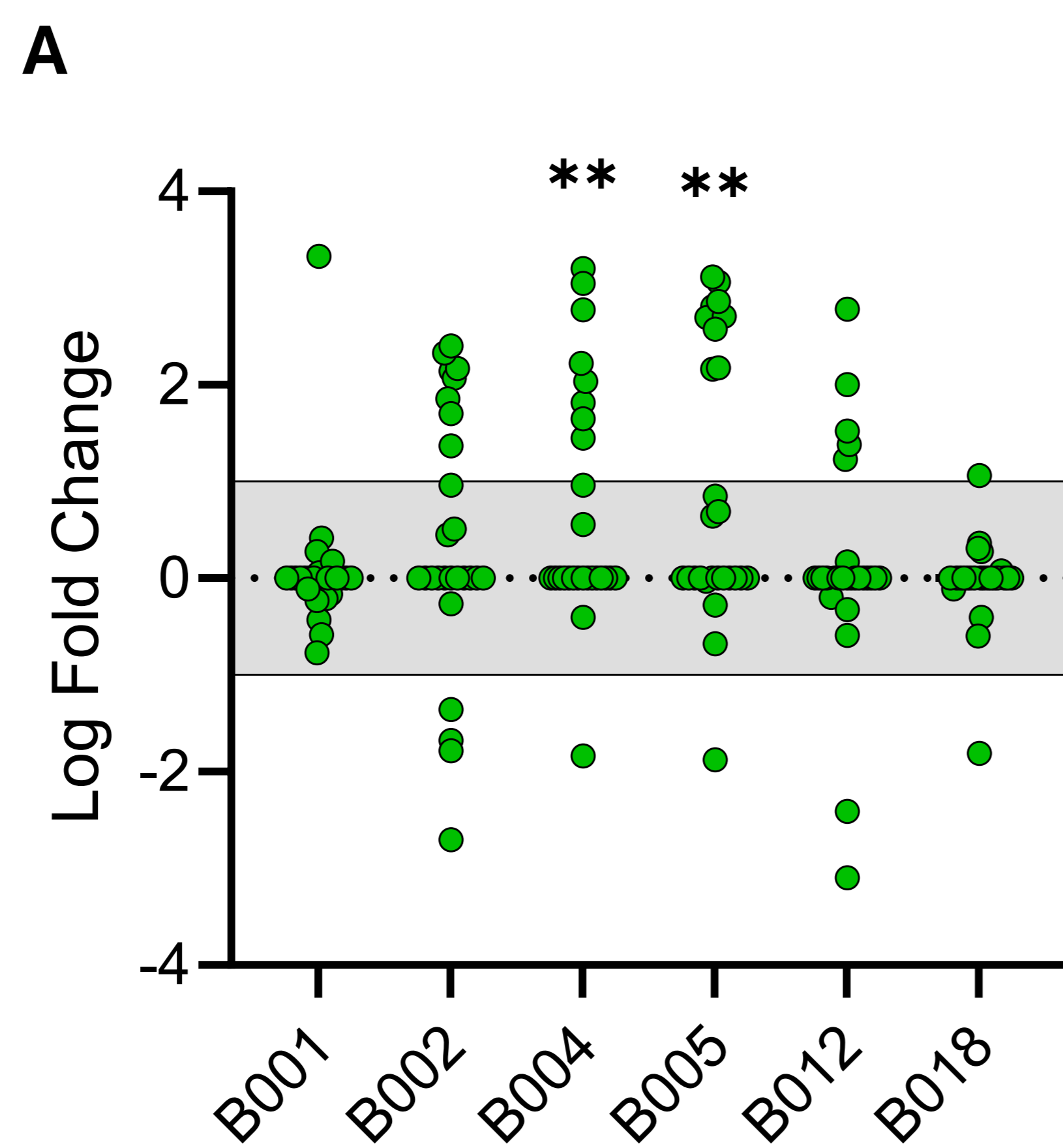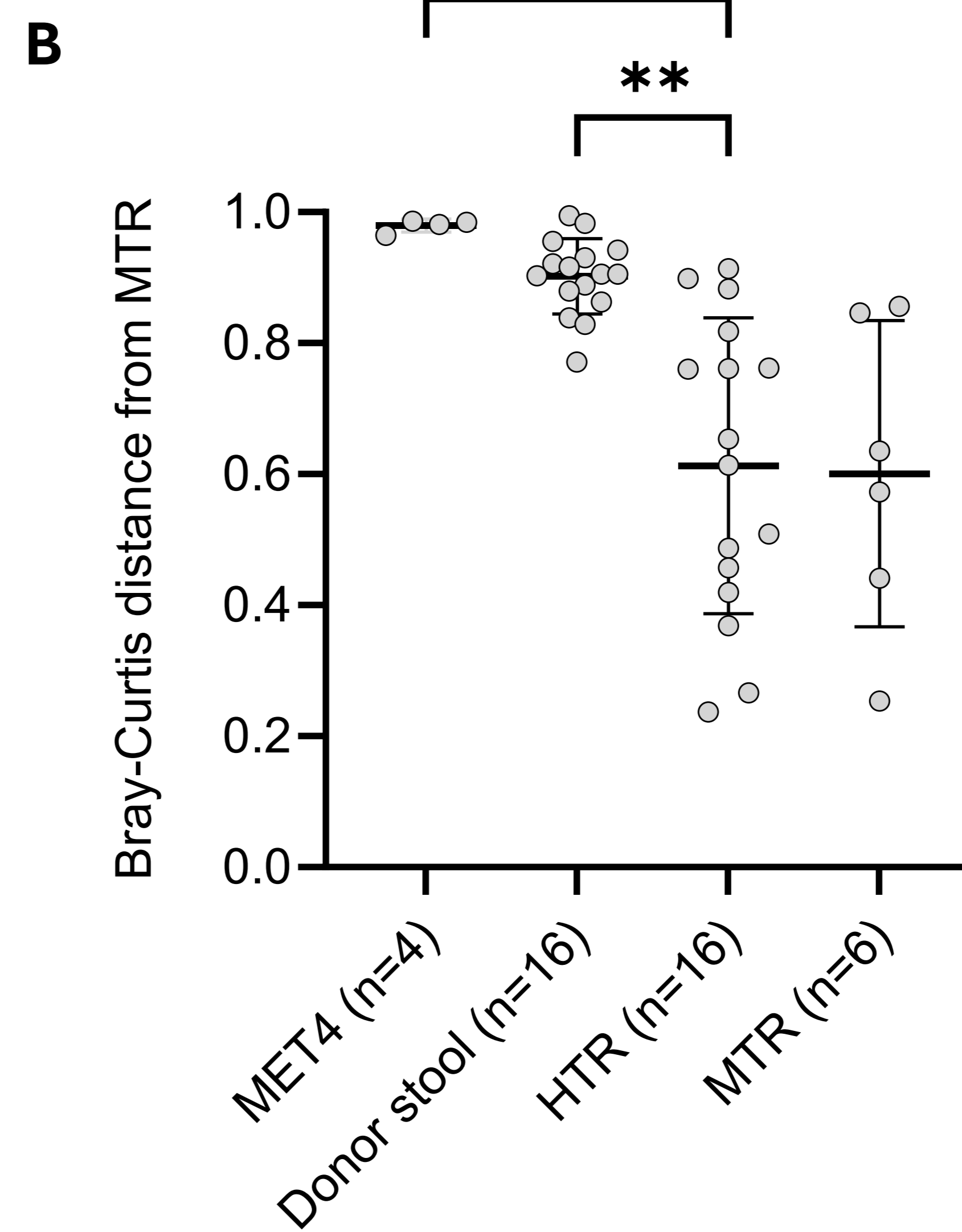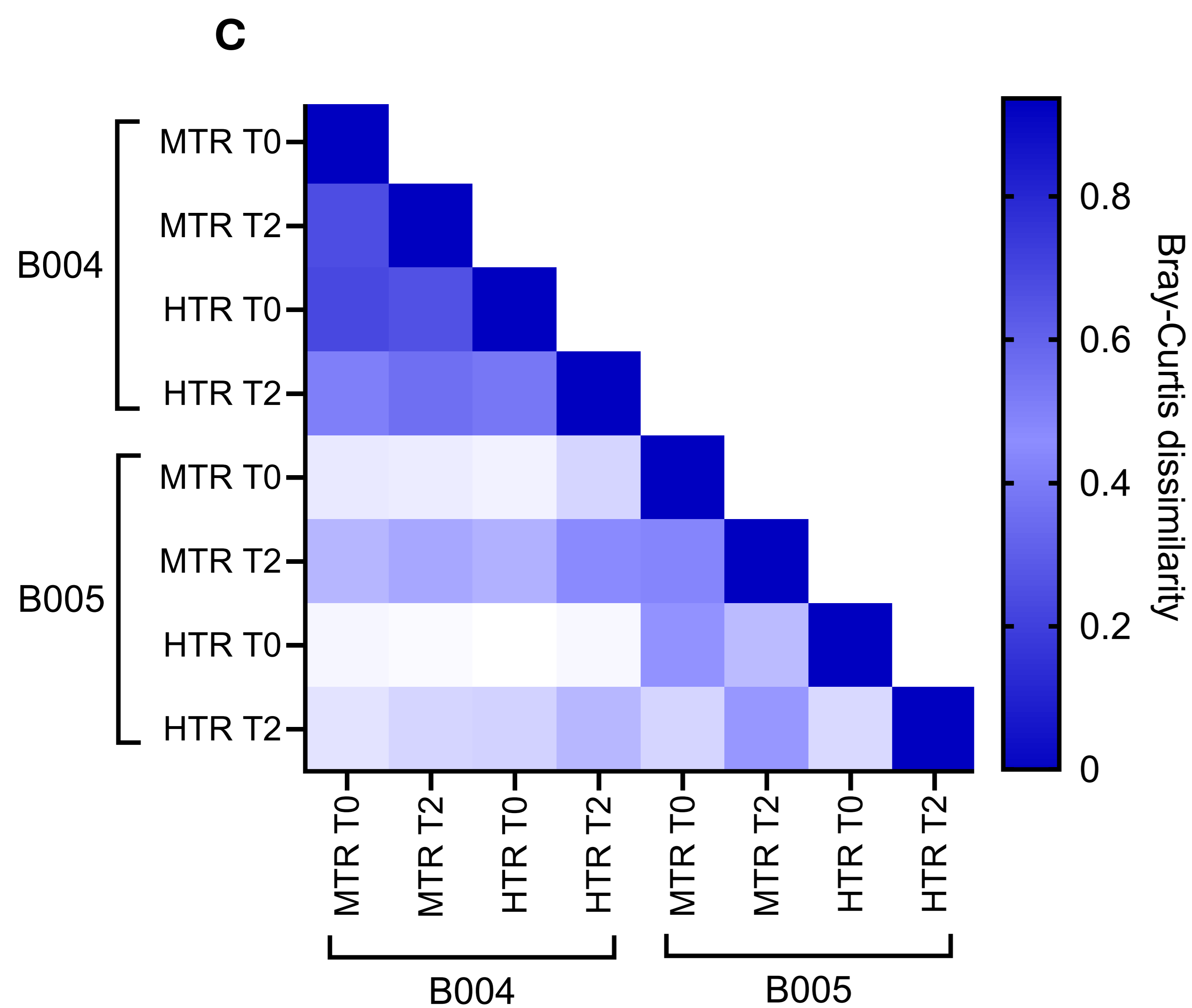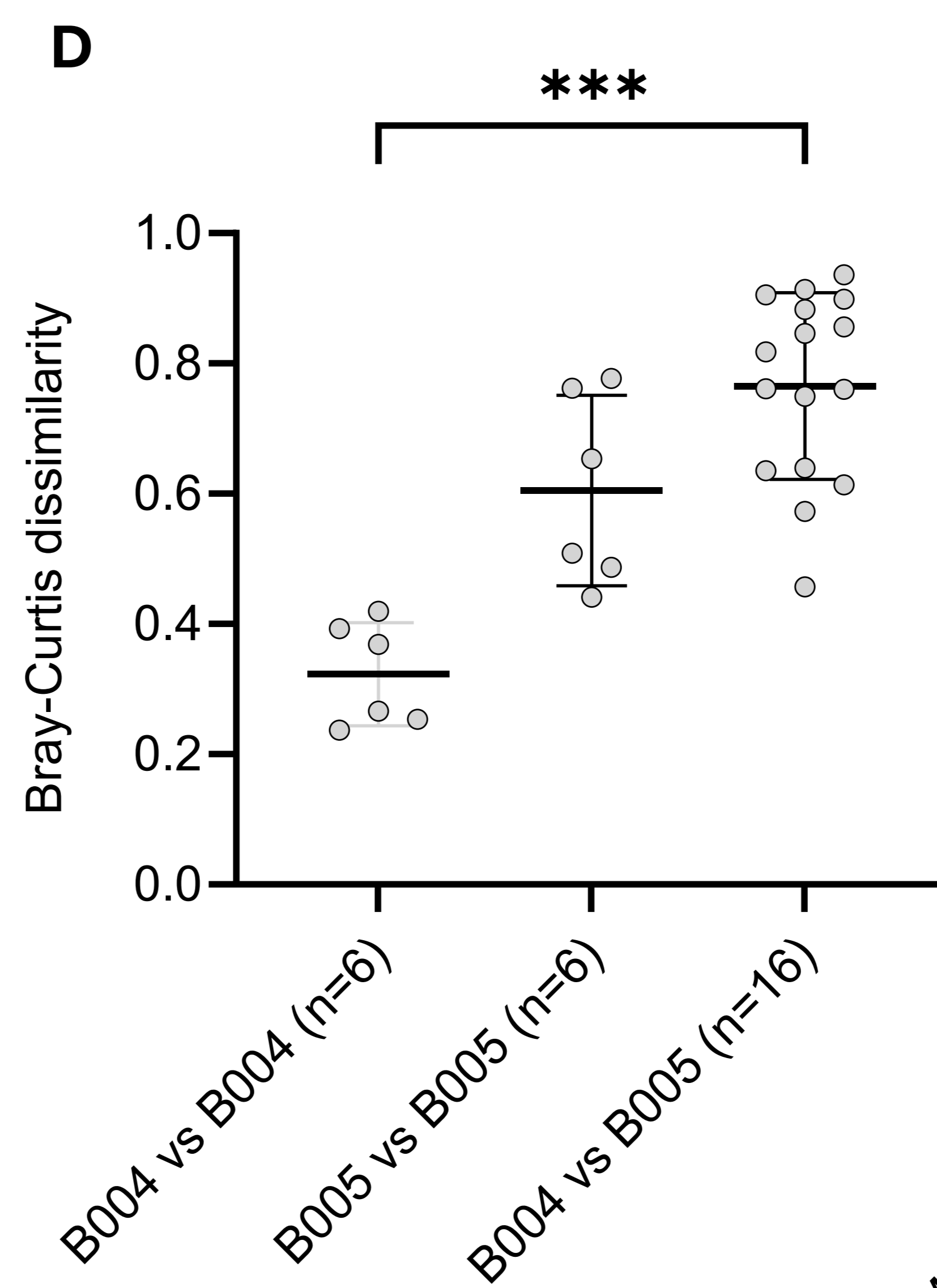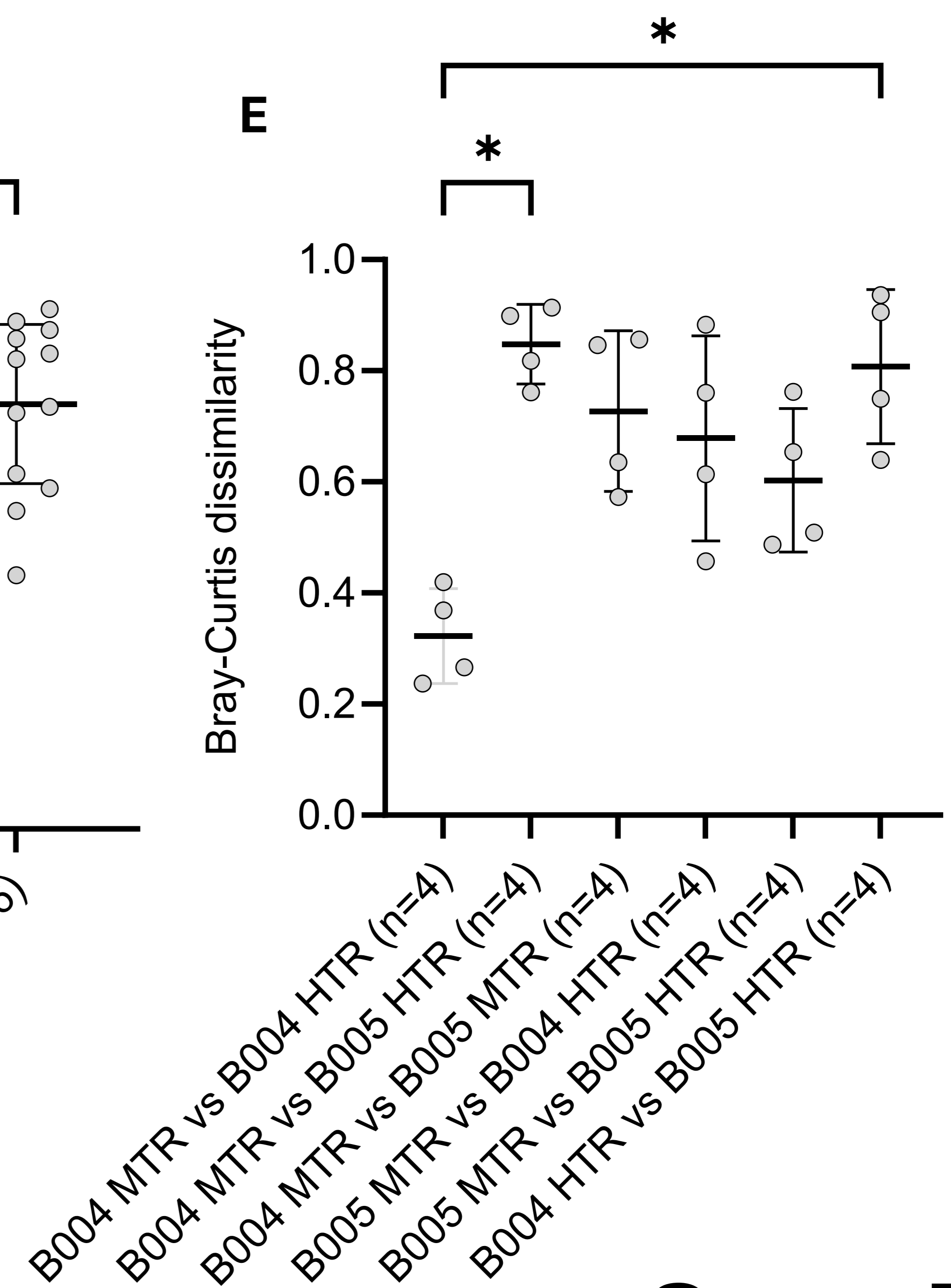

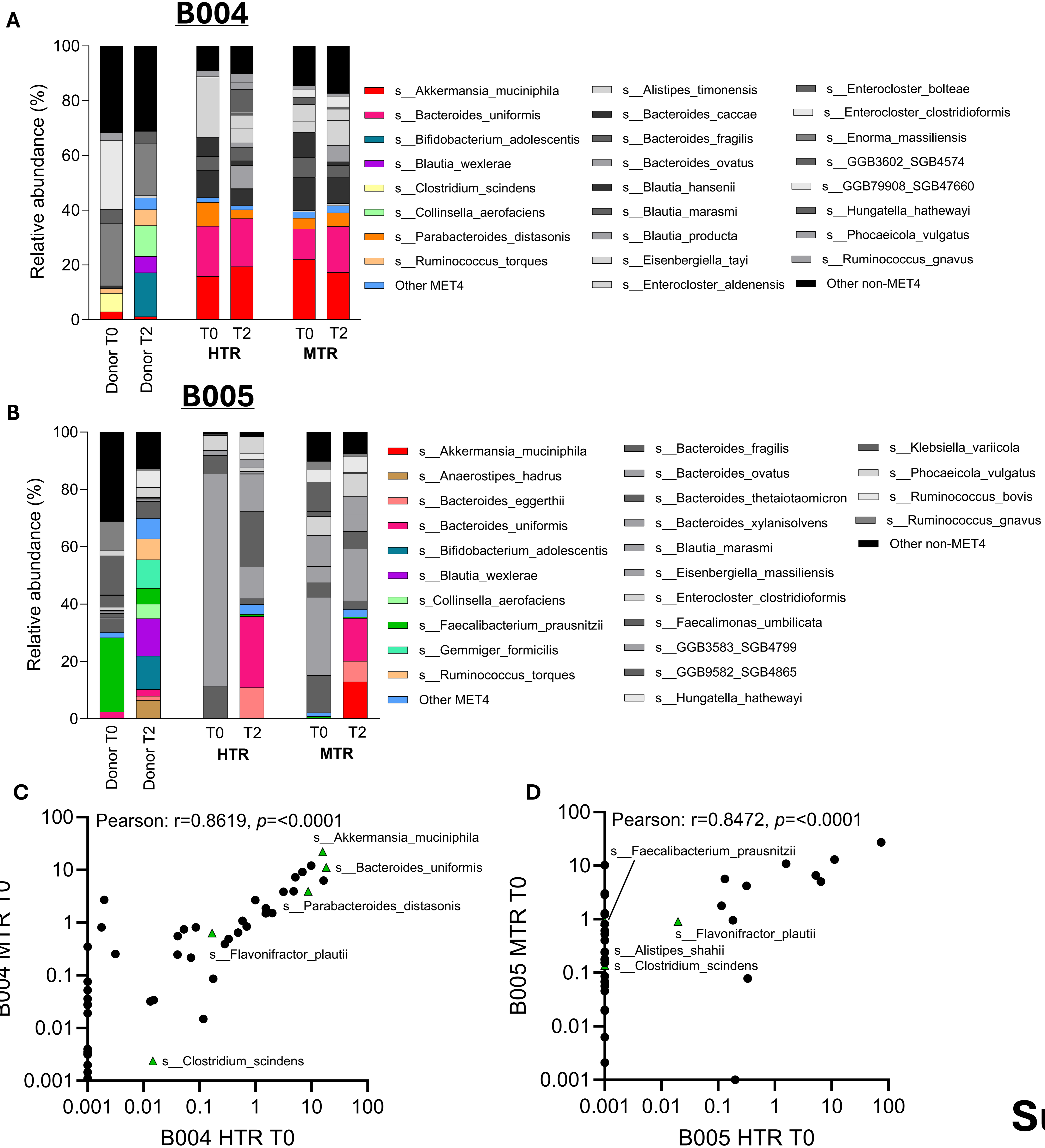

Supp Fig 7

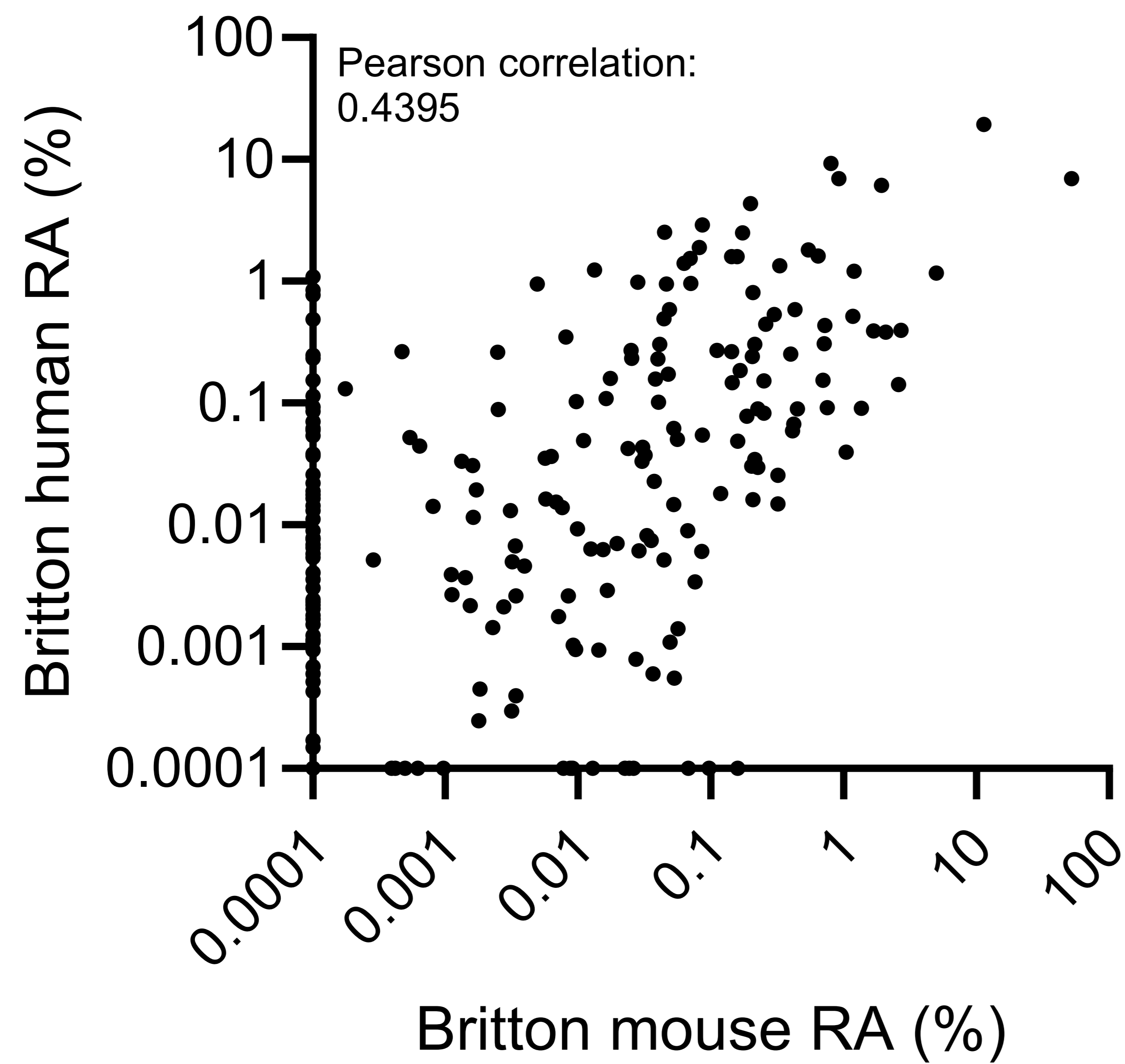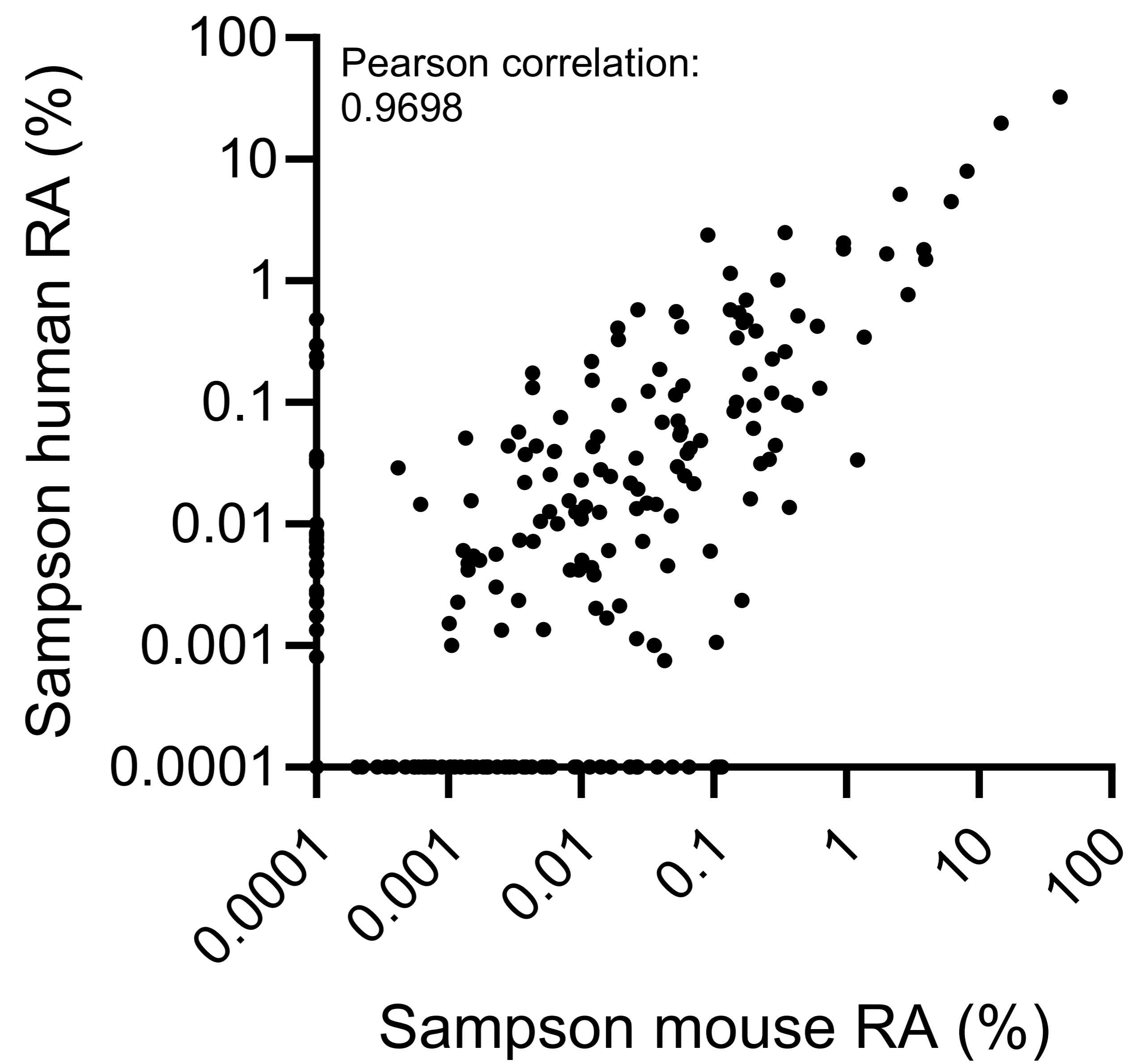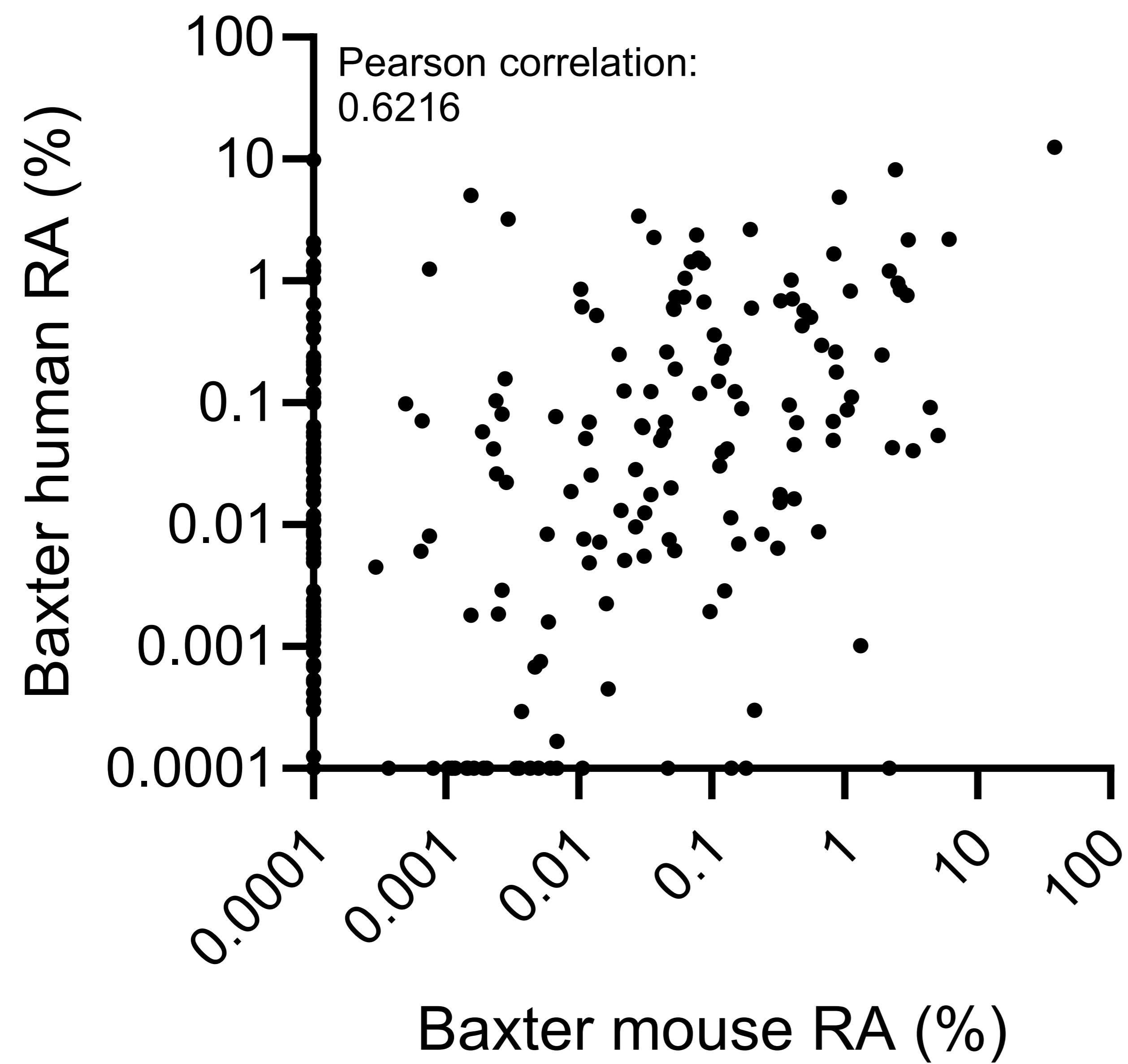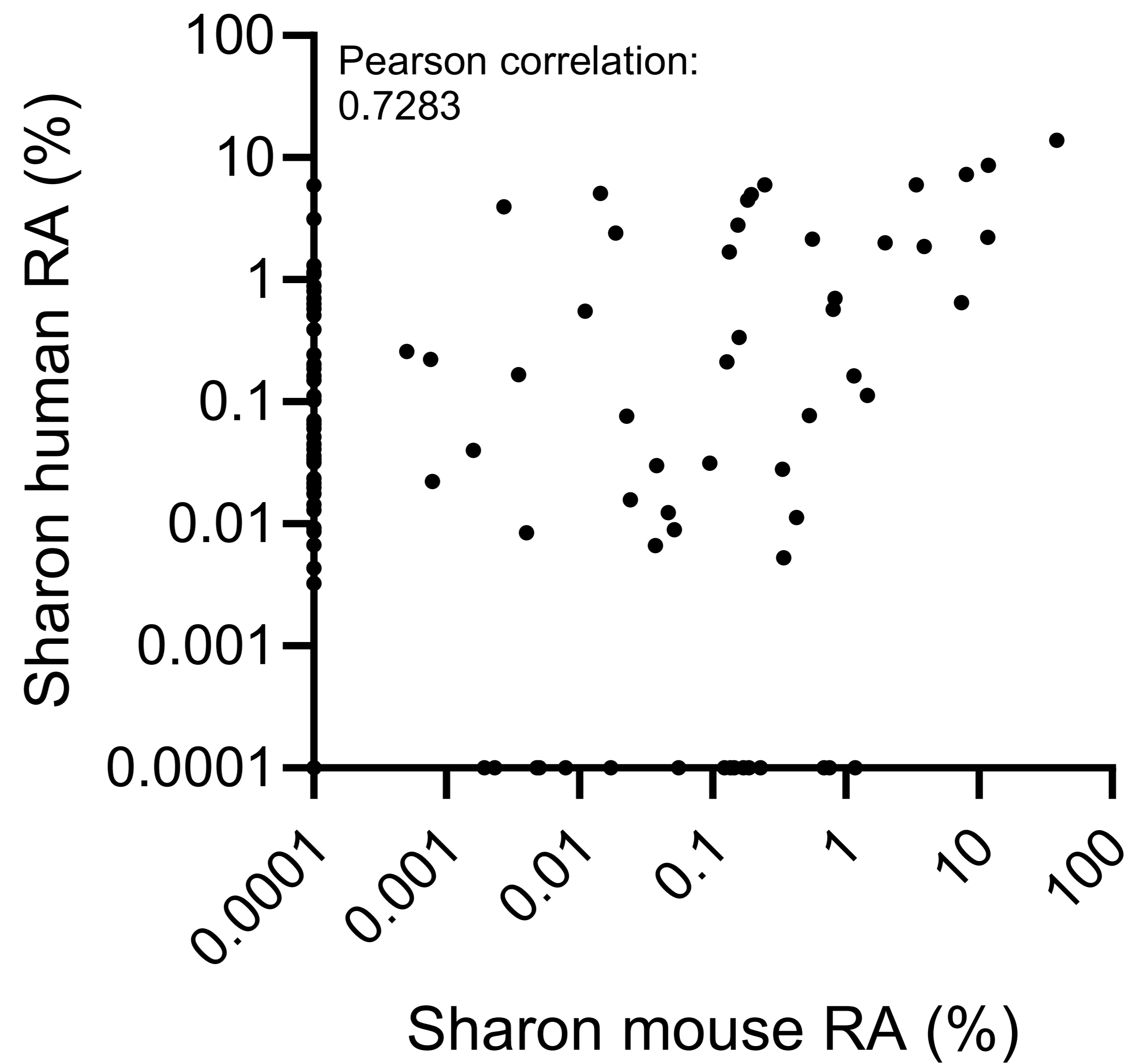

Supplement: Supplemental figures — Fig. S1 to S8. [file mbio.01904-25-s0001.pdf]
