# Supplementary material for: Assessment of ecological fidelity of human microbiome-associated mice in observational studies and an interventional trial
Source: mBio. 2025 Sep 25;16(11):e01904-25. doi: 10.1128/mbio.01904-25 (PMC12607873; doi:10.1128/mbio.01904-25)
Supplement: Supplemental legends — Supplemental figure and table legends. [file mbio.01904-25-s0002.docx]

**Supplementary Figure 1. Relative abundance histograms for human donor stools and mice receiving donor baseline and post-treatment stools**. Relative abundance (%) of phyla for human donor stool and mouse recipient stool samples. Bars from mouse samples represent the average of 2-4 mice receiving stool from the same donor.

**Supplementary Figure 2. PCoA of Bray-Curtis dissimilarity with stratification by human donor.** Principal coordinate analysis plot stratifying samples based on donor. Each dot represents an individual human donor sample or an average of mouse recipient samples from the same donor. Human donor samples are circled in blue and mouse recipient samples are circled in red.

**Supplementary Figure 3. Number of engrafting taxa in mice varies by stool and donor**. Scatter plots depicting logscale % relative abundance of taxa in germ-free mice receiving participant stools from before (T0) and after MET4 treatment (T2). Percent of taxa engrafted from donor is displayed at bottom right of each plot.

**Supplementary Figure 4. Comparison of overall functional metagenomic profiles based on host species.** PCoA plot of Bray-Curtis dissimilarities generated with functional gene pathway relative abundances. Host species comparison performed with PERMANOVA.

**Supplementary Figure 5. Enrichment of functional metagenomic pathways based on host species.** Associations between gene pathways and host species (i.e., human vs mouse) obtained with MaAsLin2. Only pathways that were significantly associated with host species (FDR-adjusted q < 0.10) were included.

**Supplementary Figure 6. Bray-Curtis dissimilarity between routes of MET4 exposure and inoculum.** (A) Log fold change of relative abundance from T0 to T2 for MET4 taxa in donor stools. Areas outside of grey box show taxa with >1 log fold increase or decrease post-treatment. One sample t-tests were performed to assess non-zero fold change. (B) Comparison of Bray-Curtis dissimilarity between MET4, donor stools, and HTR/MTR mouse stools compared to MTR mouse stools. (C) Bray-Curtis dissimilarity heat map between different routes of exposure for B004 and B005 at T0 and T2 timepoints. (D-E) Comparisons of Bray-Curtis dissimilarity between B004 and B005 mice (D) and between intra- and inter-donor routes (E). Each dot in (B, D-E) represents the Bray-Curtis dissimilarity between mice generated from a single donor stool for each inter-route/timepoint/donor comparison. Lines represent mean and whiskers represent the standard deviation in all figures. Kruskall-Wallis followed by Dunn’s multiple comparison test performed for (B, D-E). *=*p*<0.05; **=*p*<0.01; ***=*p*<0.001; ****=*p*<0.0001.

**Supplementary Figure 7. Full relative abundance histograms for mice receiving donor FMT and MET4 and scatter plots for routes at baseline T0.** (A-B) Average relative abundance (%) of stool for B004 (A) and B005 (B) from baseline (T0) and post-MET4 (T2) timepoints for human-treated route (HTR) and mouse-treated route (MTR) mice. Non-MET4 taxa are shown in grayscale. “Other” consists of taxa with <5% relative abundance in every sample. (C-D) Scatter plots showing logscale % relative abundance of gut taxa in B004 (C) and B005 (D) mice compared between routes of exposure at baseline T0. Each point represents a different taxon. MET4 taxa are represented by green triangles.

**Supplementary Figure 8. Correlation scatter plots between human and mouse relative abundance in mega-analysis studies**. Scatter plots depicting logscale average % relative abundance of taxa in HMA mice compared to their respective donors. Each dot represents an individual taxon. Pearson correlation performed for each donor-recipient comparison; all comparisons were *p*<0.0001*.*

**Supplementary Table 1. MET4 taxonomic annotations**Mega-analysis annotations only include taxa that reached the cut-off threshold (>4 total mice engrafted)

**Supplementary Table 2. Commonly engrafting taxa in HMA mouse studies.**Cut-off for inclusion in analysis was >4 total mice engrafted across studies.

**Supplementary Table 3. Engrafting MET4 taxa in consortium-exposed HMA mice.**
